# The Place of the Proctologist in a Diagnostic Group

**Published:** 1917-12

**Authors:** Alfred J. Zobel

**Affiliations:** San Francisco, Cal.


### ABSTRACTS.

### THE PLACE OF THE PROCTOLOGIST IN A DIAGNOSTIC GROUP.

**By Alfred J. Zobel, M.D., F.A.C.S., San Francisco, Cal.**

Dr. Zobel in his address stated that the Charter Members of the American Proctologic Society were the pioneer teachers and practitioners of modern proctology. Through the efforts of the Fellows of the Society proctology has received recognition as a specialty both from the American Medical Association and the American College of Surgeons.

Attention was again called to the fact that but few of the undergraduate medical schools give any adequate instructions in entereo-proctology by qualified men. In sharp contrast to this, it is noted that in every postgraduate medical school in the country there is a department of rectal and colonic surgery, whose existence is amply justified not only by the number of patients which it treats, but by the large attendance of postgraduate students.

It is urged that the undergraduate medical schools, particularly those attached to universities, should take heed of the demands and needs of modern medicine, and that they should begin to realize that failure to impart instruction in entereo-proctology, and in the other recognized specialties which have arisen in late years, impairs their standing as thorough teaching institutions.

Up to about twenty years ago all the medical needs of a community were attended to by the so-called family doctor. But year after year changes have been going on until today is the day of the specialist.

This era of specialism gives evidence of the advancement and betterment of the whole profession; it means far more efficent service rendered to the public than it has received in the past.

It now requires a close and almost undivided attention to that subject alone if one wishes to keep abreast with what is being accomplished in any special line of work.

With more knowledge and longer experience the specialist better realizes the close relationship existing between his particular field and all the other parts of the body. He further learns that while, from devoting his entire attention to his own special work, he excels therein, he consequently lacks knowledge, experience and adeptness in that of others. A a result, lately among progressive men there has arisen a movement to form what is known as "Diagnostic Groups." Group diagnosis is not a new idea. It has been used for years in the postgraduate schools, which are the only institutions possessing a staff of clinicians in every specialty of medicine and surgery. The present movement is simply an elaboration and an extension of the original idea.

Every diagnostic group should include specialists in every branch of medicine and surgery. In it should be an enteroproctologist with training and experience sufficient to warrant the interpretation of his findings being considered of some value.

The Fellows of the American Proctologic Society have repeatedly urged the necessity for co-operation with the internist, surgeon and other specialists. They have again and again pointed out that anal, rectal and colonic lesions often give rise reflexly to symptoms which may be wrongly attributed to disease in other parts of the body, and vice versa. This is especially true with regard to the reproductive and urinary organs of the male and female. Therefore, in the consideration of cases presenting symptoms in these parts, it is equally important to secure the opinions of the gynecologist, urologist; and proctologist before a correct and final diagnosis can be deduced.

It is the high-class men among the various specialists of medicine and surgery who best know the value of, and mostly insist upon the need for ano-recto-colonic examinations. They are the ones who best understand that through long and varied experience, skill in the use of the illuminated pneumatic sigmoidoscope, and ability to correctly interpret what is seen, the entero-proctologist is the one who should be relied upon to do this part in the diagnostic scheme.

The time has already arrived when even the laity recognize this, and they are now quick to take cognizance of the neglect of their medical adviser to secure for them an expert examination of the rectum and colon.

Every diagnostic group should include a competent proctologist. Only then will it be worthy of the name of "Diagnostic Group."

